# Genome-Resolved Metagenomics Reveals Distinct Phosphorus Acquisition Strategies between Soil Microbiomes

**DOI:** 10.1128/msystems.01107-21

**Published:** 2022-01-11

**Authors:** Xingjie Wu, Christopher Rensing, Dongfei Han, Ke-Qing Xiao, Yuexiu Dai, Zhixi Tang, Werner Liesack, Jingjing Peng, Zhenling Cui, Fusuo Zhang

**Affiliations:** a College of Resources and Environmental Sciences, National Academy of Agriculture Green Development, Key Laboratory of Plant-Soil Interactions, Ministry of Education, China Agricultural University, Beijing, China; b National Observation and Research Station of Agriculture Green Development, Quzhou, Hebei, China; c Institute of Environmental Microbiology, College of Resource and Environment, Fujian Agriculture and Forestry University, Fuzhou, China; d Institute of Environment and Sustainable Development in Agriculture, Chinese Academy of Agricultural Sciences, Beijing, China; e School of Earth and Environment, University of Leeds, Leeds, United Kingdom; f State Key Laboratory of Vegetation and Environmental Change, Institute of Botany, Chinese Academy of Sciences, Beijing, China; g Research Group Methanotrophic Bacteria and Environmental Genomics/Transcriptomics, Max Planck Institute for Terrestrial Microbiology, Marburg, Germany; Lawrence Berkeley National Laboratory

**Keywords:** phosphorus, *gcd*, genome, microbiome, metagenomics, MAGs, phosphorous

## Abstract

Enhancing soil phosphate solubilization is a promising strategy for agricultural sustainability, while little is known about the mechanisms of how microorganisms cope with differing phosphorus availability. Using a combination of genome-resolved metagenomics and amplicon sequencing, we investigated the microbial mechanisms involved in phosphorus cycling under three agricultural treatments in a wheat-maize rotation system and two natural reforestation treatments. Available soil phosphorus was the key factor shaping bacterial and fungal community composition and function across our agricultural and reforestation sites. Membrane-bound quinoprotein glucose dehydrogenase (PQQGDH) and exopolyphosphatases (PPX) governed microbial phosphate solubilization in agroecosystems. In contrast, genes encoding glycerol-3-phosphate transporters (*ugpB*, *ugpC*, and *ugpQ*) displayed a significantly greater abundance in the reforestation soils. The *gcd* gene encoding PQQGDH was found to be the best determinant for bioavailable soil phosphorus. Metagenome-assembled genomes (MAGs) affiliated with *Cyclobacteriaceae* and *Vicinamibacterales* were obtained from agricultural soils. Their MAGs harbored not only *gcd* but also the *pit* gene encoding low-affinity phosphate transporters. MAGs obtained from reforestation soils were affiliated with *Microtrichales* and *Burkholderiales*. These contain *ugp* genes but no *gcd*, and thereby are indicative of a phosphate transporter strategy. Our study demonstrates that knowledge of distinct microbial phosphorus acquisition strategies between agricultural and reforestation soils could help in linking microbial processes with phosphorus cycling.

**IMPORTANCE** The soil microbiome is the key player regulating phosphorus cycling processes. Identifying phosphate-solubilizing bacteria and utilizing them for release of recalcitrant phosphate that is bound to rocks or minerals have implications for improving crop nutrient acquisition and crop productivity. In this study, we combined functional metagenomics and amplicon sequencing to analyze microbial phosphorus cycling processes in natural reforestation and agricultural soils. We found that the phosphorus acquisition strategies significantly differed between these two ecosystems. A microbial phosphorus solubilization strategy dominated in the agricultural soils, while a microbial phosphate transporter strategy was observed in the reforestation soils. We further identified microbial taxa that contributed to enhanced phosphate solubilization in the agroecosystem. These microbes are predicted to be beneficial for the increase in phosphate bioavailability through agricultural practices.

## INTRODUCTION

Phosphorus availability is essential for soil health and plant growth (1, 2). Unlike nitrogen, soil phosphorus exists in many organic and inorganic forms, and most of these forms are physically immobilized by soil minerals that are not readily bioavailable for utilization by plants (3, 4). While plant growth and biomass accumulation are constrained by soil phosphorus bioavailability, mitigating phosphorus limitation in an agroecosystem has been shown to be a promising strategy for sustainable phosphorus resource management and crop production in the future (5). Microbes have great potential to increase soil phosphorus availability and are the key players in regulating phosphorus transformation processes (6–8). A series of microbially released enzymes (e.g., phytase, C-P lyase, and phosphonatase) and organic acids (e.g., gluconic acid, malic acid, and oxalic acid) are able to solubilize recalcitrant phosphorus (6, 9). The soil microbiome also contributes to plant phosphorus uptake by arbuscular mycorrhizal symbiosis (10). The balance between microbial phosphorus assimilation and scavenging processes further regulates soil phosphorus bioavailability (11). Despite the importance of microbially mediated phosphorus cycling to terrestrial ecosystem functioning and services, little has been known about the microorganisms involved in phosphate solubilization and their genetic potential to adapt to phosphorus limitation.

Microbial genes involved in the soil phosphorus cycle encode four distinct functional traits: inorganic phosphate solubilization (e.g., *gcd*, *ppa*, and *ppx*), organic phosphorus mineralization (e.g., *phoA* and *phoD*), transporters (e.g., *pit*, *pstA*, *pstB*, and *ugpQ*), and regulatory genes (e.g., *phoB* and *phoR*) (12–15). Among these genes, the *gcd* gene encoding the membrane-bound quinoprotein glucose dehydrogenase (PQQGDH; EC.1.1.5.2) is the major determinant of rock phosphate and hydroxyapatite solubilization by gluconic acid production (16, 17). Exopolyphosphatase (PPX), encoded by the *ppx* gene, was shown to play an important role in the degradation of inorganic polyphosphate into phosphate (18). Microbes also possess efficient phosphorus uptake systems to effectively compete for soil phosphorus resources with other biota (19, 20). The genes encoding the high-affinity phosphate-specific transporter (*pst*) and low-affinity inorganic phosphate transporter (*pit*) are essential to facilitate microbial phosphorus uptake under phosphorus-depleted or phosphorus-rich conditions (21). A positive correlation between the abundance of microbial genes encoding high-affinity phosphorus uptake and low bioavailability of soil phosphorus has been reported (20). Phosphate-solubilizing microorganisms have previously been characterized as plant growth-promoting bacteria, including strains of the genera *Bacillus*, *Burkholderia*, Pseudomonas, and *Rhizobium* (22–24). A recent genome-resolved metagenome study expanded our knowledge of phosphate-solubilizing capabilities from *Firmicutes* and *Proteobacteria* to members of other phyla, including *Acidobacteria*, *Bacteroidetes*, *Gemmatimonadetes*, and *Planctomycetes* (13). However, the link between their phosphorus acquisition strategies, including inorganic phosphate solubilization, and particular soil conditions still needs to be further elucidated.

A meta-analysis of research data from 192 samples showed us that soil available phosphorus (AP) significantly differs between agricultural and forest soils across China (see Fig. S1 in the supplemental material). To understand the microbial mechanisms underlying the different phosphorus availability in these two ecosystems, we combined functional metagenomics and amplicon sequencing (bacterial 16S rRNA gene and fungal internal transcribed spacer [ITS] region). Given that key traits linked to nutrient acquisition can be inferred from genomic data (25), we anticipated that this approach would allow us to obtain more comprehensive profiles of microbial community composition and function (26) and thus to elucidate the genetic potential for phosphorus acquisition in soils under contrasting management practices. Located in the North China plain, our experimental field site involved three agricultural treatments (no fertilizer, chemical fertilizer, and chemical fertilizer plus manure) in a wheat-maize rotation system and two natural reforestation treatments initiated 12 and 42 years ago. We specifically aimed to investigate microbially mediated inorganic phosphate solubilization and phosphate transport mechanisms in agricultural and reforestation soils and to determine the driving factors for regulating microbial phosphate solubilization. In particular, we hypothesized that soil microbiomes inhabiting resource-limited reforestation soils exhibit efficient phosphate transport systems to compete for and acquire available phosphorus with minimal energy investment. In contrast, the high-resource inputs into agricultural soils stimulate the activity of microbes involved in inorganic phosphate solubilization with more energy investment in extracellular metabolic processes.

10.1128/mSystems.01107-21.1 FIG S1 Meta-analysis showing the content of total phosphorus (TP) and bioavailable phosphorus (AP) in 120 agricultural soils (maize) and 72 forest soils sampled across various soil types and climatic zones in China. Download FIG S1, TIF file, 1 MB. Copyright © 2022 Wu et al. 2022 Wu et al. https://creativecommons.org/licenses/by/4.0/ This content is distributed under the terms of the Creative Commons Attribution 4.0 International license.

## RESULTS

### Soil properties.

**FIG 1:**
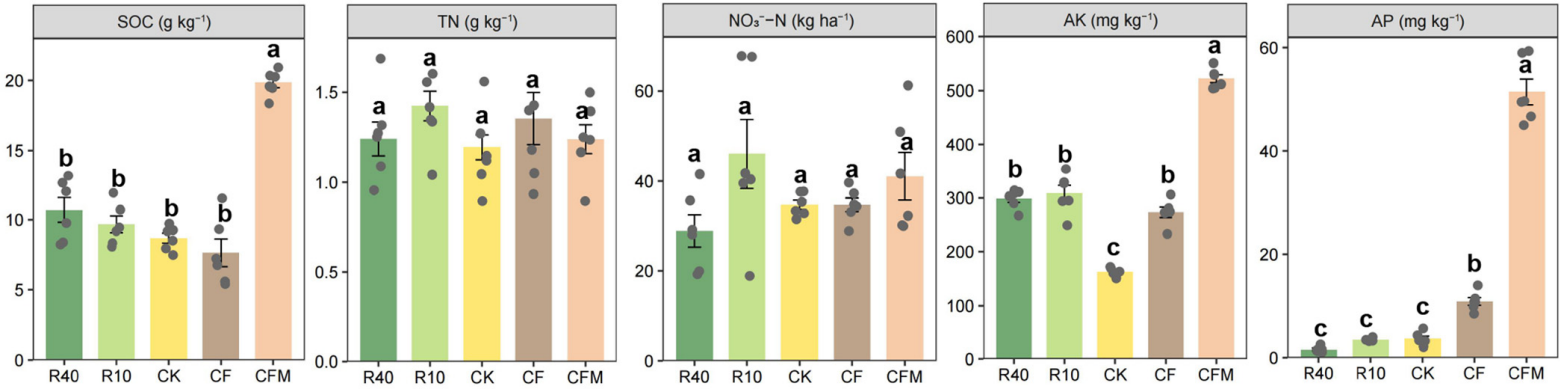
Soil chemical properties in the agricultural and reforestation soils. The effects of the different management practices on SOC, TN, NO_3_^−^-N, AK, and AP are shown. Different letters indicate significant differences between treatments (*P < *0.05).

Reforestation significantly decreased soil available phosphorus (AP) contents in comparison to agricultural soil (*P < *0.05) (Fig. 1). The lowest soil AP content (1.53 mg kg^−1^) was observed under R40 treatment, while the highest soil AP content (51.50 mg kg^−1^) was found under CFM (chemical fertilizer plus organic manure) treatment. The soil organic carbon (SOC) content was the highest (19.85 g kg^−1^) under CFM treatment, which was directly enhanced by organic manure inputs (*P < *0.05) (Fig. 1). Soil available potassium (AK) was significantly increased by 111.17 and 359.67 mg kg^−1^ under chemical fertilizer (CF) and CFM treatments compared to CK (control without fertilization) treatment (*P < *0.05) (Fig. 1). R40 and R10 significantly increased soil AK content by 136.50 and 145.67 mg kg^−1^ in comparison to CK treatment, respectively (*P < *0.05) (Fig. 1).

### Treatment-driven changes in taxonomic and functional profiles.

**FIG 2:**
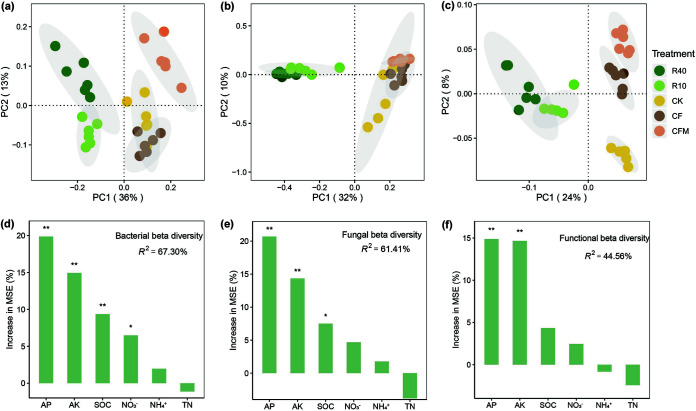
Microbial diversity and the effects of soil parameters on the microbial diversity in agricultural and reforestation soils. Shown are the results from principal-coordinate analysis (PCoA) of bacterial (a), fungal (b), and functional gene (c) diversity. The analyses are based on 16S rRNA gene (bacteria), ITS region (fungi), and gene profiles at KEGG level 4. Random forest analysis was applied to determine the contribution of each soil property to bacterial (d), fungal (e), and functional gene (f) diversity. The importance of each predictor was determined by the increase in mean squared error (MSE). Asterisks indicate that the predictor was significant (*, *P < *0.05; **, *P < *0.01).

Principal-coordinate analysis (PCoA) revealed distinct clustering between agricultural and reforestation soils (Fig. 2). Reforestation explained 36 and 32% of the total bacterial and fungal diversity variation in the first axis, respectively (Fig. 2a and b). Bacterial rather than fungal diversity varied with reforestation age. With regard to the functional gene profiles (KEGG level 4), reforestation accounted for 24 and 8% of the variation in the first and second axes, respectively (Fig. 2c). Random forest analysis further showed that soil properties explained 67.30, 61.41, and 44.56% of bacterial, fungal, and functional beta diversity variation, respectively (Fig. 2d to f). Among the soil chemical parameters, AP was the most important predictor for bacterial, fungal, and functional beta diversity, followed by AK and SOC (Fig. 2d to f). Soil AP was positively correlated to both taxonomic and functional beta diversity (*P < *0.05) (see Fig. S2a in the supplemental material).

10.1128/mSystems.01107-21.2 FIG S2 (a) Correlation between microbial functional gene diversity (KEGG) indices (alpha and beta diversity) and soil chemical properties. Alpha diversity was represented by the Shannon index, while the beta diversity was calculated by NMDS. Asterisks indicate significant correlations based on regression analysis. A Venn plot displays the distribution of microbial indicators across different treatments (b). The red rectangle represents the indicators that were shared in treatments of reforestation and agricultural ecosystems, respectively. (c and d) Co-occurrence pattern (c) and node degree (d) of microbial indicators that are representative of reforestation and agricultural ecosystems. Spearman correlations between OTUs were calculated, and only robust and significant correlations were included (ρ > 0.80; *P_FDR_* < 0.05). Download FIG S2, TIF file, 2 MB. Copyright © 2022 Wu et al. 2022 Wu et al. https://creativecommons.org/licenses/by/4.0/ This content is distributed under the terms of the Creative Commons Attribution 4.0 International license.

### Correlation between microbial indicators and soil available phosphorus.

**FIG 3:**
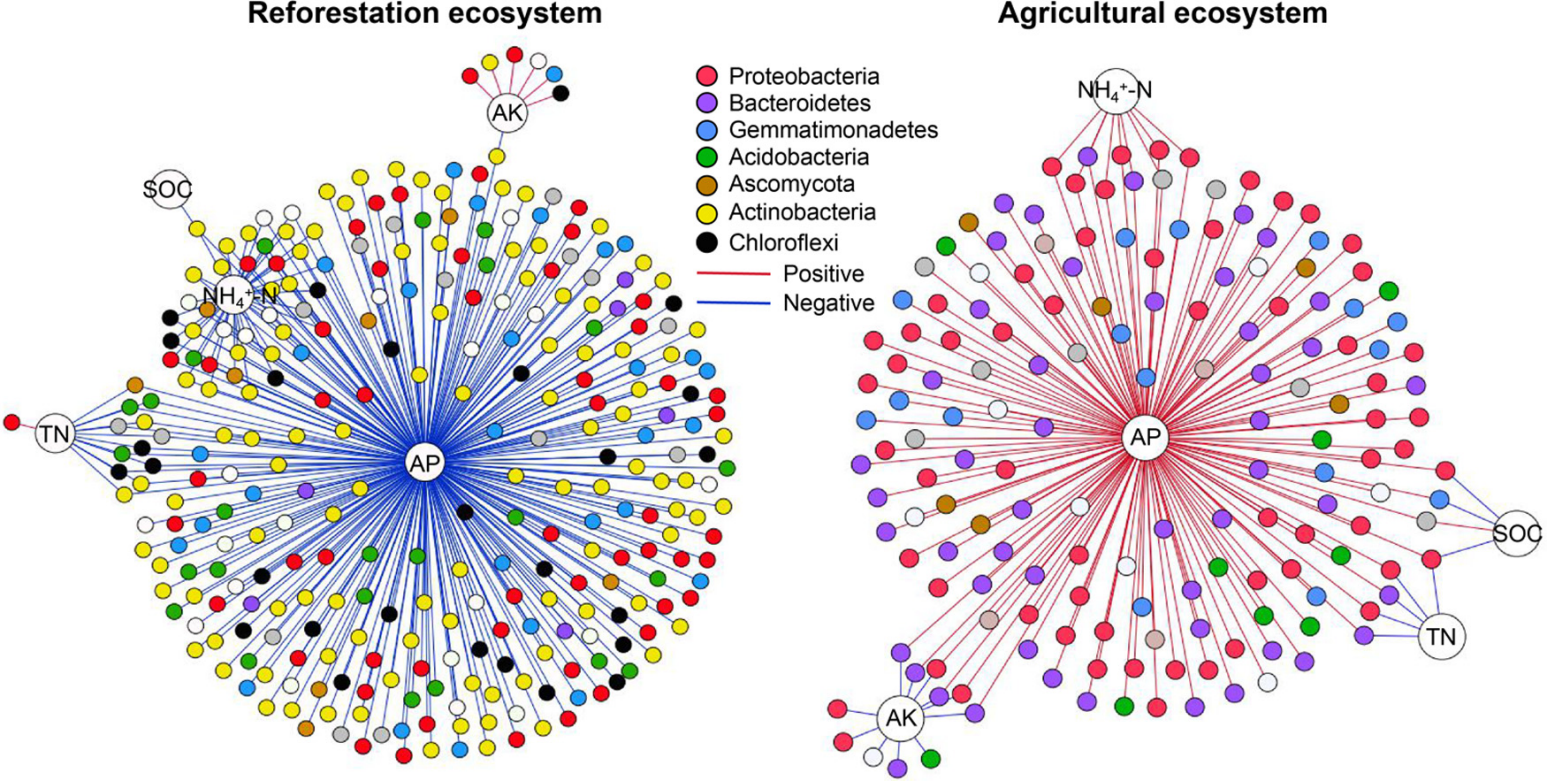
Microbial indicators in agricultural and reforestation soils. The indicators shared by R40 and R10 treatments were viewed as the representative microbial indicators for reforestation soils, and indicators shared by CK, CF, and CFM treatments were viewed as the representative microbial indicators for agricultural soils. A significant correlation between microbial indicators and soil properties was calculated by Spearman correlation analysis (*P < *0.05) and visualized in Gephi. The positive and negative correlations between microbial indicators are shown in red and blue, respectively.

Soil AP was found to be the key soil variable affecting microbial indicators (Fig. 3). A total of 181 microbial indicators were shared by the CK, CF, and CFM treatments in the agricultural soils, while 324 microbial indicators were shared by the R40 and R10 treatments in the reforestation soils (see Fig. S2b and Tables S1 and S2 in the supplemental material). Co-occurrence networks showed that the microbial indicators were closely connected and formed distinct clusters between reforestation and agricultural soils (Fig. S2c and d). Soil AP was identified to be significantly positively correlated to most of the microbial indicators (86.7%) in agricultural soils, while it was significantly negatively correlated to most of the microbial indicators (90.1%) in reforestation soils (Fig. 3). At the phylum level, *Actinobacteria* and *Chloroflexi* were mainly indicative of reforestation soils, while *Bacteroidetes* was representative of agricultural soils (Fig. 3; Table S2). At the family level, *Chitinophaceae* (*Bacteroidetes*), *Sphingomonadaceae* (*Alphaproteobacteria*), and *Xanthomonadaceae* (*Gammaproteobacteria*) were abundant indicators for the agricultural soils, while members of the *Solirubrobacteraceae* (*Thermoleophilia*) were indicative of the reforestation soils and negatively correlated with soil AP (see Fig. S3 and Table S2 in the supplemental material). Among fungi, *Hypocreales* and *Eurotiales* (*Ascomycota*) were identified to be the indicators of agricultural and reforestation soils, respectively.

10.1128/mSystems.01107-21.3 FIG S3 Correlation between soil AP and the normalized abundance of microbial orders (*Chitinophagales*, *Cytophagales*, *Sphingomonadales*, *Xanthomonadales*, *Myxococcales*, and *Solirubrobacterales*) (a) and families (*Chitinophagaceae*, *Cyclobacteriaceae*, *Sphingomonadaceae*, *Xanthomonadaceae*, *Myxococcaceae*, and *Solirubrobacteraceae*) (b) based on amplicon sequencing of the 16S rRNA gene. Download FIG S3, TIF file, 1.9 MB. Copyright © 2022 Wu et al. 2022 Wu et al. https://creativecommons.org/licenses/by/4.0/ This content is distributed under the terms of the Creative Commons Attribution 4.0 International license.

10.1128/mSystems.01107-21.6 TABLE S1 The number and total abundance of differentially abundant OTUs (enriched and depleted) across the different treatments. The abundance of OTUs that significantly varied between different treatments was calculated using the package *edgeR* with an FDR-corrected value of *P*_FDR_ < 0.05. Download Table S1, TIF file, 0.2 MB. Copyright © 2022 Wu et al. 2022 Wu et al. https://creativecommons.org/licenses/by/4.0/ This content is distributed under the terms of the Creative Commons Attribution 4.0 International license.

10.1128/mSystems.01107-21.7 TABLE S2 Normalized abundance of microbial indicators shown for the agricultural and reforestation soils at the phylum, order, and family levels. Download Table S2, TIF file, 0.5 MB. Copyright © 2022 Wu et al. 2022 Wu et al. https://creativecommons.org/licenses/by/4.0/ This content is distributed under the terms of the Creative Commons Attribution 4.0 International license.

### Correlation between abundance of phosphorus cycling genes and soil available phosphorus.

**FIG 4:**
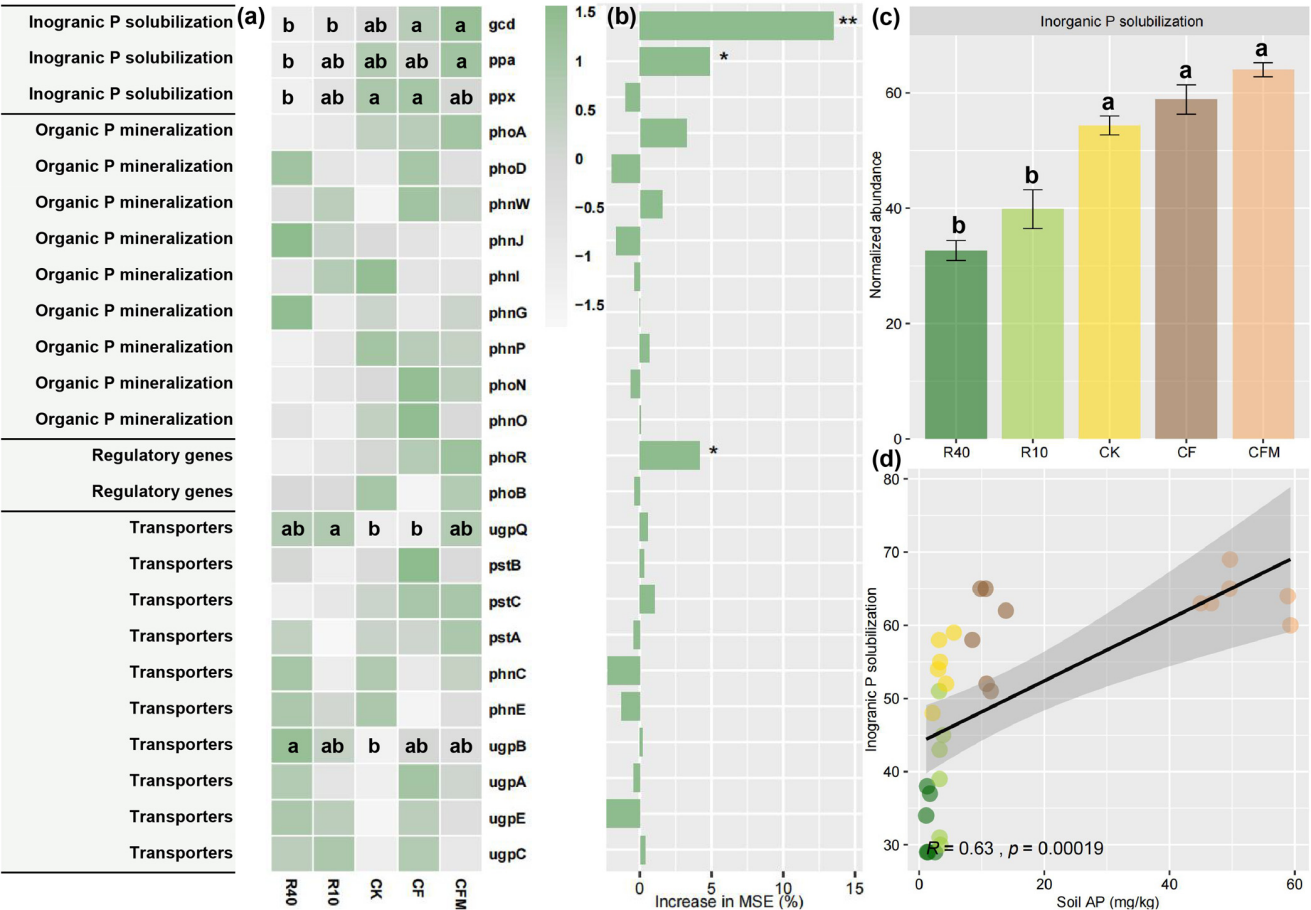
Abundance of phosphorus transformation-related genes in agricultural and reforestation soils. (a) Heat map displaying the Z-scored transformed abundance of 24 microbial phosphorus transformation-related genes based on metagenomic data. Predictors for soil available phosphorus were determined by random forest analysis. The importance of each predictor was evaluated by the increase in mean squared error (MSE). Asterisks indicate that the predictor was significant (*, *P < *0.05; **, *P < *0.01). (b) Bar plot showing the collective abundance of *gcd*, *ppa*, and *ppx* across the five treatments. (c) The three genes determine the capacity for soil inorganic phosphate solubilization. Different letters indicate significant differences between particular treatments. (d) Correlation between soil available phosphorus (AP) and genetic potential for soil inorganic phosphate solubilization.

**FIG 5:**
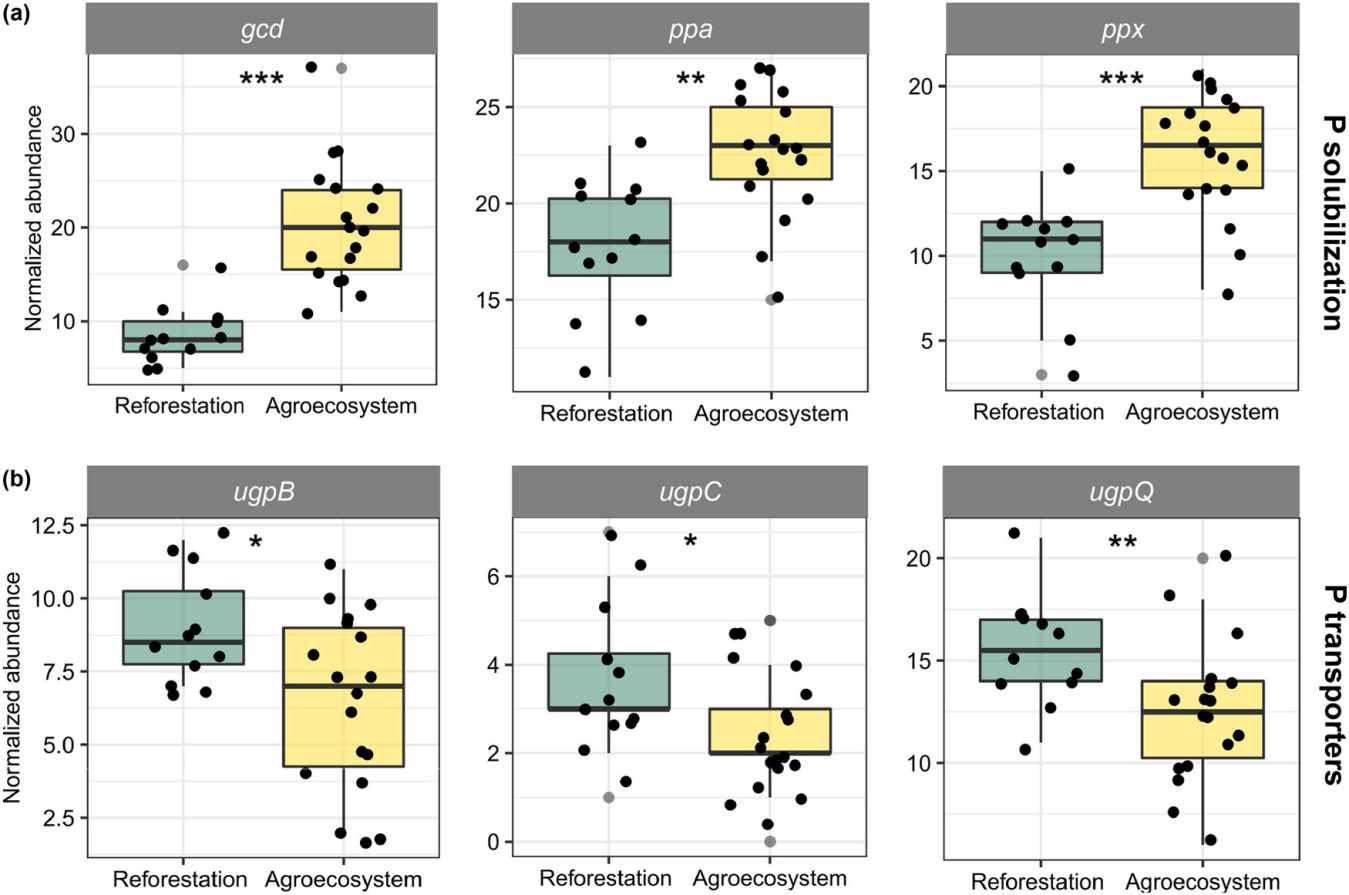
The normalized abundance of the *gcd*, *ppa*, and *ppx* genes (phosphate solubilization) as well as *ugpB*, *ugpC*, and *ugpQ* genes (glycerol-3-phosphate transporter) in agricultural and reforestation soils. Asterisks indicate significant difference in gene abundance between the two ecosystems (*, *P < *0.05; ***, *P < *0.001).

A total of 24 genes encoding functions in phosphorus metabolism were detected (Fig. 4a; see Table S3 in the supplemental material). PCoA analysis of these genes showed distinct clusters in reforestation and agricultural soils (see Fig. S4 in the supplemental material). Random forest analysis showed that the *gcd* gene was the most important predictor for soil AP, followed by *ppa* and *phoR* (*P < *0.05) (Fig. 4b; see Table S4 in the supplemental material). The abundance of genes encoding functions involved in inorganic phosphate solubilization was the highest in the agricultural soils (Fig. 4c). In addition, their collective abundance (*gcd*, *ppa*, and *ppx*) was positively associated with soil AP and significantly enriched in the agricultural soils (Fig. 4d and 5a; Fig. S4). However, the *ugpB*, *ugpC*, and *ugpQ* genes, which encode a glycerol-3-phosphate transporter, were significantly enriched in reforestation soils (R40 and R10) (*P < *0.05) (Fig. 5b).

10.1128/mSystems.01107-21.4 FIG S4 Principal-coordinate analysis (PCoA) of microbial genes that encode functions involved in phosphorus cycling (a). (b to d) Correlations between soil AP and normalized abundance of the *gcd* (b), *ppa* (c), and *ppx* (d) genes. Download FIG S4, TIF file, 1.7 MB. Copyright © 2022 Wu et al. 2022 Wu et al. https://creativecommons.org/licenses/by/4.0/ This content is distributed under the terms of the Creative Commons Attribution 4.0 International license.

10.1128/mSystems.01107-21.8 TABLE S3 Genes encoding P cycle-related functions, which were identified in our metagenomic samples. Download Table S3, TIF file, 0.7 MB. Copyright © 2022 Wu et al. 2022 Wu et al. https://creativecommons.org/licenses/by/4.0/ This content is distributed under the terms of the Creative Commons Attribution 4.0 International license.

10.1128/mSystems.01107-21.9 TABLE S4 Identification of 455 PQQ-GCD proteins in our 30 metagenomic samples using InterProScan. Download Table S4, XLSX file, 0.02 MB. Copyright © 2022 Wu et al. 2022 Wu et al. https://creativecommons.org/licenses/by/4.0/ This content is distributed under the terms of the Creative Commons Attribution 4.0 International license.

**FIG 6:**
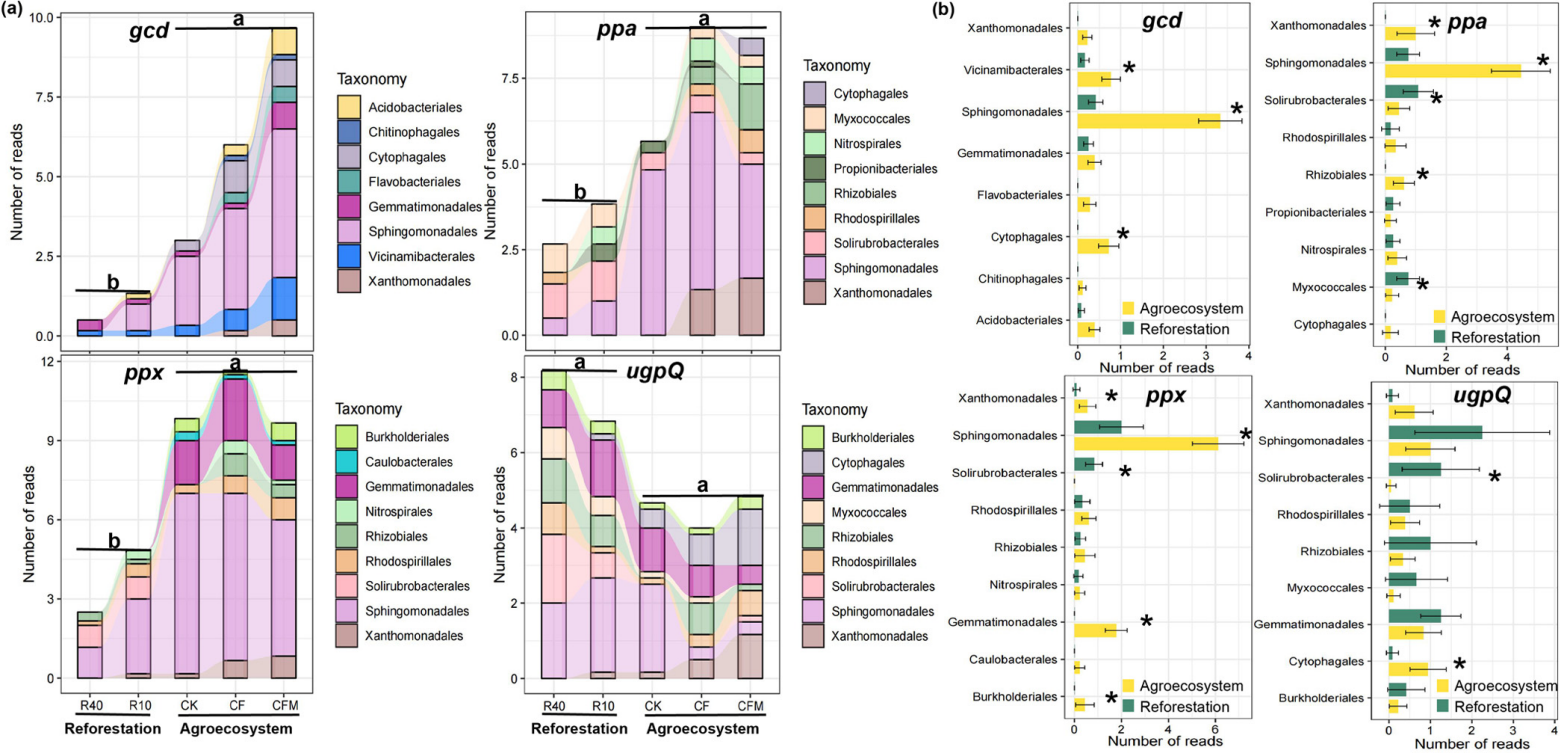
Taxonomic assignments of the *gcd*, *ppa*, *ppx* and *ugpQ* genes at the order level. (a) The bar plot displays the number of reads that are assigned to the *gcd*, *ppa*, *ppx*, and *ugpQ* genes. (a and b) The taxonomic assignment of the *gcd*, *ppa*, *ppx*, and *ugpQ* genes (a) and their abundance in agricultural and reforestation soils (b). Different letters in panel a (a and b) represent significant difference in the normalized read numbers obtained from the agricultural and reforestation soils, respectively.

The taxonomic compositions of key genes involved in phosphorus cycling (*gcd*, *ppa*, *ppx*, and *ugpQ*) significantly differed between the two ecosystems (Fig. 6). In particular, the relative abundance of *gcd* genes affiliated with *Cytophagales*, *Sphingomonadales*, and *Vicinamibacterales* was significantly greater in the agricultural soils than in the reforestation soils (*P < *0.05) (Fig. 6). Likewise, *ppa* and *ppx* genes affiliated with *Solirubrobacterales*, *Sphingomonadale*s, and *Xanthomonadales* were significantly enriched in the agricultural soils (*P < *0.05) (Fig. 6). In contrast, *ugpQ* genes affiliated with *Cytophagales* and *Solirubrobacterales* showed a significantly greater abundance in the reforestation soils than in the agricultural soils (*P < *0.05) (Fig. 6).

### Linking MAGs to phosphorus acquisition strategy.

**FIG 7:**
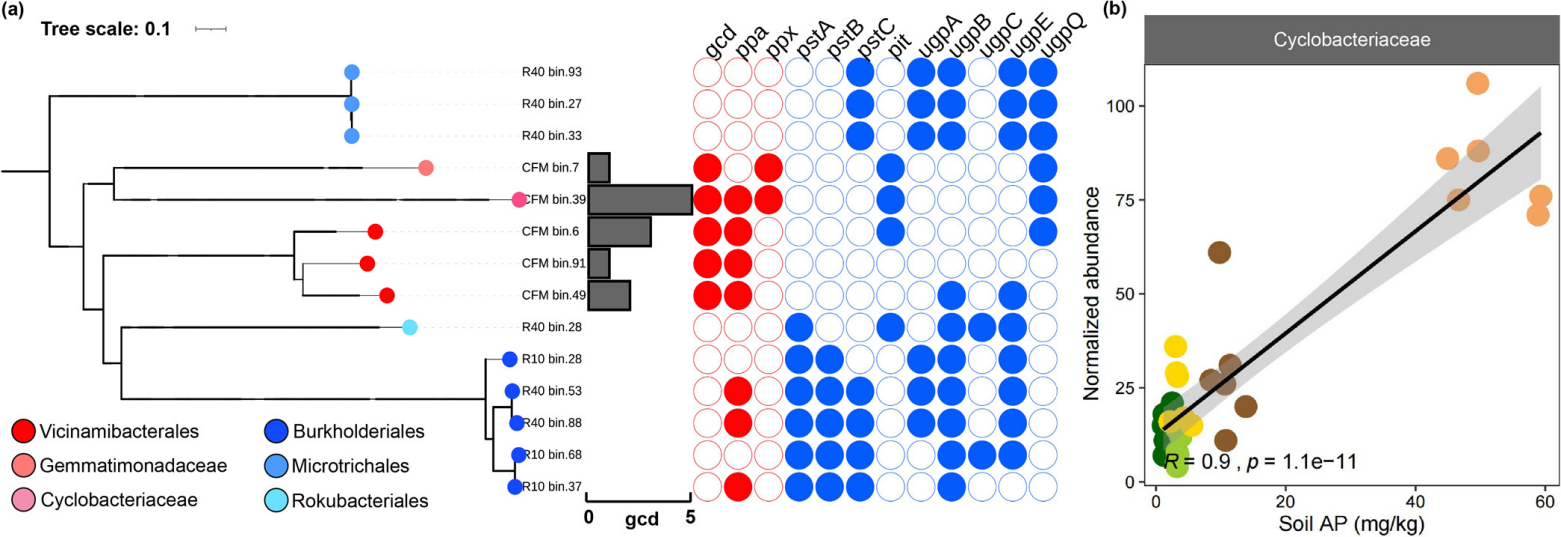
(a) Maximum likelihood tree of 14 MAGs based on a concatenated alignment of 120 marker genes from GTDB-Tk. The phylogenetic tree was visualized in the i-Tol platform. The bar plot displays the *gcd* gene copy numbers in our 14 MAGs, while the heat map shows the abundance of phosphorus transformation-related genes among the 14 MAGs. The taxonomic affiliation of the MAGs is based on GTDB-Tk classification. (b) Correlation between *Cyclobacteriaceae* abundance in the metagenomic data sets and soil available phosphorus (AP) content.

The 14 metagenome-assembled genomes (MAGs) had an average completeness and contamination of 81.1% and 3.9%, respectively (see Table S5 in the supplemental material). The five *gcd*-containing MAGs from agricultural soils were phylogenetically classified as *Cyclobacteriaceae* (*Bacteroidetes*), *Gemmatimonadaceae* (*Gemmatimonadetes*), and *Vicinamibacteria* (*Acidobacteria*) (Fig. 7a; Table S5). All of these five *gcd*-containing MAGs harbored at least one *ppa* or *ppx* gene. Most of the MAGs contained one to three *gcd* genes. However, MAG CFM_bin.39 contained five *gcd* genes, one *ppa* gene, and two *ppx* genes. This MAG was obtained from phosphorus-rich agricultural soil and belongs to the *Cyclobacteriaceae*. Among transporters, MAG CFM_bin.39 only encoded the low-affinity phosphate transporter (*pit*) but no high-affinity phosphate transporters (*ugp*, *pst*). The *Cyclobacteriaceae* family was highly abundant under CFM treatment and positively correlated to soil AP content (*R* = 0.9, *P < *0.001) (Fig. 7b). In addition, the *Vicinamibacterales* (CFM_bin.6) harbored three *gcd* genes and one *pit* transporter gene, but no high-affinity *ugp* or *pst* transporter system. These two genomes were representative of the phosphate solubilization strategy in the agroecosystem.

10.1128/mSystems.01107-21.10 TABLE S5 Genome size, GC content, completeness, contamination, phylum-level assignment, the number of *gcd* genes of 14 MAGs, and distribution of *phzF* (K06998) and *phzS* (K20940) among the MAGS obtained in our study. Download Table S5, TIF file, 0.18 MB. Copyright © 2022 Wu et al. 2022 Wu et al. https://creativecommons.org/licenses/by/4.0/ This content is distributed under the terms of the Creative Commons Attribution 4.0 International license.

The MAGs obtained from the phosphorus-deficient R10 and R40 treatments displayed genes encoding a high-affinity phosphate transporter (*pst*) and the glycerol-3-phosphate transporter (*ugp*). For instance, the MAGs affiliated with *Rokubacteriales* (R40_bin.28), *Microtrichales* (*Acdimicrobila*) (R40_bin.93, R40_bin.33, and R40_bin.27) and *Burkholderiales* (*Proteobacteria*) (R10_bin.28, R10_bin.37, R10_bin.68, R40_bin.88, and R40_bin.53) harbored genes encoding the glycerol-3-phosphate transporter (*ugpA*, *ugpB*, and *ugpE*), while no *gcd* genes could be detected in these MAGs (Fig. 7a). In addition, three MAGs in the reforestation soils contained a gene involved in phenazine biosynthesis (*phzS*) (Table S5). Phenazine is a newly characterized redox-active antibiotic that has been shown to be involved in phosphorus acquisition under phosphorus limitation (8).

## DISCUSSION

In this study, we analyzed the distinct responses of soil microbial communities to soil AP in two contrasting ecosystems by using a combination of genome-resolved binning, comparative metagenomics, amplicon sequencing, and multivariate analysis. Notably, the genetic potential of the retrieved MAGs confirmed that the phosphorus acquisition strategies in reforestation and agricultural soils significantly differ. Thus, our study expands the knowledge of phosphate-solubilizing capacities and microbial adaptive traits to soil microbiomes in reforestation and agricultural soils.

### Microbial indicators and functional responses driven by soil available phosphorus.

Soil AP was the most important predictor for microbial diversity and structure across the reforestation and agricultural soils. Our study showed that soil AP shaped microbial functional diversity, which reinforced the view that phosphorus bioavailability shaped microbial functions (9). In addition to the association between microbial functional diversity and soil AP, significant correlations between microbial indicators and soil AP were observed in both reforestation and agricultural soils (Fig. 2 and 3). In particular, *Chitinophagales*, *Cytophagales*, *Sphingomonadales*, and *Xanthomonadales* were the order-level indicators that positively correlated to soil AP in agricultural soils (Fig. S3 and Table S2). These taxa may be potential indicators that regulate soil phosphorus bioavailability, as supported by the results of our meta-analysis correlating taxon abundance with bioavailable phosphorus across 120 agricultural soils and 72 forest soils in China (*P < *0.001) (see Fig. S5 in the supplemental material). Our results were further confirmed by MAGs being affiliated with *Chitinophagales*, *Cytophagales*, and *Sphingomonadales*, which have previously been reported to have the genetic potential to solubilize phosphate (13, 27–29). Furthermore, the taxonomic assignments of *gcd* reads to *Chitinophagales*, *Cytophagales*, *Sphingomonadales*, and *Xanthomonadales* strengthens the above findings on the taxon-specific distribution of phosphate-solubilizing capacities among bacteria (Fig. 6).

10.1128/mSystems.01107-21.5 FIG S5 A meta-analysis of order-level taxa identified in our study to be positively (*Chitinophagales*, *Cytophagales*, *Sphingomonadales*, and *Xanthomondales*) or negatively (*Solirubrobacterales*) correlated with soil AP. (a) Their abundance in 120 agricultural soils (maize) and 72 forest soils is shown in relation to bioavailable phosphorus. (b) The taxon abundances were calculated using 16S rRNA gene sequence data sets published together with the soil AP measurements. (c) Conceptual diagram illustrating the differing phosphorus acquisition strategies of microbiomes in agricultural and reforestation soils. Download FIG S5, TIF file, 2.9 MB. Copyright © 2022 Wu et al. 2022 Wu et al. https://creativecommons.org/licenses/by/4.0/ This content is distributed under the terms of the Creative Commons Attribution 4.0 International license.

### The *gcd* gene is the determinant predictor of soil AP.

Phosphate solubilization was found to be the most essential and significant microbial functional trait in various soils, due to the prevalence of phosphate in complex inorganic and organic forms that may be inaccessible (4, 30). Correspondingly, the PQQGDH enzyme was shown to be the most important indicator for inorganic phosphate solubilization in microbial communities (6, 20). In our study, the *gcd* gene encoding PQQGDH was found to be a prominent and significant predictor for soil AP and microbial phosphate solubilization capacity in both agricultural and reforestation soils, which is consistent with previous results obtained for microbiomes in postmining soils (13). However, the abundance of the *gcd* gene varied up to 3-fold between reforestation and agricultural soils. This is striking as previous studies have found that, regardless of the C:N:P stoichiometry differences, the microbial potential to solubilize phosphate is highly stable (7, 31). An increase in *gcd* gene abundance does not necessarily provide final proof for an increase in the phosphate solubilization activity of microbial communities. However, the strong positive correlation between *gcd* gene abundance and soil AP provides additional evidence that phosphorus bioavailability is regulated by microbial phosphate solubilization (Fig. S4). The high importance of the *gcd* gene as a biomarker for soil phosphorus cycling is reasonable given that inorganic phosphate solubilization has been proposed to be a dominant process in phosphorus-rich soils (13, 20).

### Functional traits in phosphorus metabolism.

Microbial phosphorus transformation processes are regulated by soil phosphorus bioavailability (9, 20). The microbial genes encoding phosphorus cycling in the reforestation and agricultural soils indicate distinct metabolic pathways of uptake of and competition for phosphorus resources. Soil microorganisms have a number of physiological strategies to access phosphorus nutrients, including production of organic acids to solubilize recalcitrant forms of phosphorus and efficient phosphate transport systems (32). Indeed, the microbial communities residing in agricultural soil were shown to invest in inorganic phosphate solubilization by organic acid synthesis (*gcd*) or enzymatic activity (*ppa* and *ppx*). We define these traits as a phosphate solubilization strategy. Thus, in the agricultural soils, this bacterial strategy was governed by the bioavailability of soil phosphorus, which was positively correlated to the abundance of genes encoding phosphate solubilization. In contrast, the microbial genes encoding the glycerol-3-phosphate transporter (*ugpB*, *ugpC*, and *ugpQ*) are highly indicative of a fierce competition for bioavailable phosphorus, which we refer to as a phosphate transport strategy.

In agreement with our hypotheses, microbial phosphorus acquisition in agricultural and reforestation soils can thus be attributed to two distinct strategies, namely, (i) inorganic phosphate solubilization and (ii) high-affinity transport of phosphate. Microbial phosphorus acquisition by synthesis of organic acids was shown to be a slow process and requires a major investment in metabolic resources (9). Microorganisms thriving in agricultural soils with organic fertilizer input favor the synthesis of organic acids for inorganic phosphate solubilization. This finding agrees well with previous reports on phosphate-solubilizing bacteria in agricultural soils (17, 24). Soil microbiomes are competitive for bioavailable phosphorus rather than for phosphate solubilization in resource-limited environments. This has been proposed according to the optimal foraging theory (33). To survive under unfavorable soil conditions, an efficient microbial community should acquire resources (e.g., phosphorus) with minimal energy investment by efficient transporters (34, 35). The higher abundance of genes encoding high-affinity phosphate transporters in the reforestation soils than in the agricultural soils and the negative correlation of their gene abundance to soil AP suggest that phosphorus deficiency stimulates competition for phosphorus. This may also explain why phosphorus was more depleted in R40 soils (1.53 mg kg^−1^) than in R10 soils (3.35 mg kg^−1^). Indeed, the gene abundance of *ugpB*, *ugpC*, and *ugpQ* was significantly higher in the reforestation soils than in the agricultural soils. This phosphate transporter was shown to empower the soil microbiome to efficiently compete for phosphorus resources with other biota when bioavailable phosphorus is scarce (16).

### Functional traits of MAGs between agricultural and reforestation soils.

The distinct phosphorus acquisition strategies operating in reforestation and agricultural soils were further evidenced by the specific MAGs. The taxonomic identity of the *gcd*-containing MAGs revealed that phosphate-solubilizing bacteria are associated with poorly characterized families, such as *Cyclobacteriaceae* (*Cytophagales*), *Gemmatimonadaceae* (*Gemmatimonadales*), *Vicinamibacteria* (*Vicinamibacterales*), and *Binatia* (Fig. 7). The *Cyclobacteriaceae* MAG was found to harbor as many as five *gcd* genes encoding PQQGDH (Fig. 7). These findings further corroborate the enrichment of *Cyclobacteriaceae* to agricultural soils (Fig. S3), which is consistent with a previous report that *Cyclobacteriaceae* members thrive in environments with high phosphorus content (36). In addition, there is a significantly positive correlation between *Cyclobacteriaceae* abundance and soil AP (*R* = 0.9; *P < *0.001). Thus, it is reasonable to propose that *Cyclobacteriaceae* is one of the key taxa in contributing to the solubilization and bioavailability of soil inorganic phosphorus in phosphorus-rich environments (37). We further found that *Vicinamibacterales*-associated MAGs (CFM_bin.6) harbor three *gcd* gene copies and a single *pit* gene copy, which provides evidence that certain bacteria have great phosphate solubilization capacity coupled with low-affinity transporters. The absence of genes (*pst* and *ugp*) encoding high-affinity transporter systems suggests that, compared with other biota, these bacteria have no competitive advantage in the acquisition of soil AP under phosphorus-depleted conditions, while they rely on their inorganic phosphate solubilization potential to meet the needs for cellular growth and reproduction.

In contrast, MAGs retrieved from the reforestation soils harbor genes encoding efficient phosphate uptake systems, including the glycerol-3-phosphate transporter (*ugp*). This gene pattern was confirmed by three MAGs affiliated with the *Microtrichales* (R40_bin.93, R40_bin.33, and R40_bin.27) and five MAGs affiliated with *Burkholderiales* (R10_bin.28, R10_bin.37, R10_bin.68, R40_bin.88, and R40_bin.53). *Burkholderiales* able to produce extracellular phosphatases were found to be beneficial microbes involved in phosphate solubilization (23, 24). In our study, microbial phosphorus uptake was mainly governed by glycerol-3-phosphate transporters in the reforestation soils. This observation agrees well with our finding that *ugp* genes showed a significantly higher abundance in the reforestation soils than in agricultural soils. It is thought that the *ugp*-encoded transporters facilitate bacteria to utilize alternative phosphorus substrates (e.g., phosphate esters) to cope with phosphorus starvation conditions (38). Phosphate esters, like glycerol-3-phosphate, have been shown to be acquired by microorganisms through *ugpBAECQ*-encoded transporters (39). Among the genes in the *ugp* gene cluster, *ugpQ*, encoding glycerol-3-phosphodiesterase, can further release glycerol-3-phosphate (38). Microorganisms in phosphorus-depleted reforestation soils may take up glycerol-3-phosphate as an alternative to maintain basic cellular activities. Collectively, the metabolic traits encoded by the retrieved MAGs further support a phosphate transport strategy, as we proposed for microorganisms inhabiting the reforestation soils.

### Conclusions.

The different phosphorus acquisition strategies between agricultural and reforestation soils in our field study may have site-specific reasons but are more likely due to a widely distributed land use change effect. This view is strongly supported by the results of our meta-analysis. These land use change effects are summarized in a conceptual diagram in Fig. S5c. The diagram highlights that agricultural farming practices shape soil microbiomes toward inorganic phosphate solubilization. The microbial phosphate solubilization potential was significantly greater in the agricultural soils than in the reforestation soils. *Cyclobacteriaceae* and *Vicinamibacterales* were indicative of the phosphate solubilization strategy in agricultural soils. In contrast, the major strategy employed by microorganisms in the phosphorus-depleted reforestation soils was to efficiently acquire and compete for phosphorus by high-affinity phosphate transport systems. Finally, our reconstructed MAGs improve our knowledge of the microbial mechanisms that govern the acquisition of soil AP. Our findings may help to improve phosphorus availability for sustainable agriculture by harnessing microbial communities.

## MATERIALS AND METHODS

### Study site and soil sampling.

All soil samples were taken from a National Observation and Research Station of Agriculture Green Development located in Quzhou, China (115.94°E, 36.78°N). The soil is calcareous fluvo-aquic. The farmland sampling site was a typical wheat (Triticum aestivum L.)-maize (Zea mays L.) rotation cropping system with a temperate continental monsoon climate. Two reforestation sites have been naturally restored from this wheat-maize rotation farmland since 1978 (R40) and 2008 (R10) without anthropogenic disturbance. The major plants growing in the restored forests are elms (Ulmus pumila L.). The mean annual temperature and precipitation are 13.2°C and 490 mm, respectively. Three farmland treatments were set as follows: a control without fertilization (CK), chemical fertilizer (CF), and chemical fertilizer plus organic manure (CFM) with a wheat-maize rotation system. The chemical fertilizer was applied at concentrations of 185 kg ha^−1^ nitrogen, 120 kg ha^−1^ phosphorus, and 100 kg ha^−1^ potassium in the wheat season and 185 kg ha^−1^ nitrogen, 45 kg ha^−1^ phosphorus, and 90 kg ha^−1^ potassium in the maize season. The cattle manure was amended at a rate of 12 Mg ha^−1^ each year. Soil available phosphorus in the cattle manure was 11.8 g kg^−1^.

The topsoil (2 to 20 cm) was sampled during the wheat season of May 2020. In the case of the reforestation soils, the upper litter layer (0 to 2 cm) was manually removed. Soil samples were taken in an S-type sampling trajectory, and 4 soil cores were collected and pooled to make a composite sample. A total of 30 soil composite samples were collected from 5 treatments with 6 replicates. The rocks and plant residues in the soil samples were manually removed. The fresh soil samples were immediately transported to the laboratory in an ice box. Soil samples were randomly subdivided into two parts for molecular and chemical analyses, respectively. Soil samples for molecular analysis were stored at −20°C.

### Soil chemical analyses.

Soil samples were air-dried for 2 weeks and sieved through 0.25-mm-pore mesh before chemical analysis. Soil ammonium (NH_4_^+^-N) and nitrate (NO_3_^−^-N) were extracted by 0.01 mol L^−1^ CaCl_2_ and then measured by continuous flow analysis (TRAACS 2000; Bran & Luebbe, Norderstedt, Germany). Soil available phosphorus (AP) was extracted by shaking at 200 rpm for 30 min with 0.50 mol L^−1^ NaHCO_3_ (40). Soil available potassium (AK) was extracted by 1 mol L^−1^ CH_3_COONH_4_ and then measured by flame photometry. Soil organic carbon (SOC) and total nitrogen (TN) were measured by CN analyzer after treatment with 1 mol L^−1^ HCl to remove carbonate (Elementar, Langenselbold, Germany).

### DNA extraction and amplicon sequencing.

DNA was extracted from 2 g of soil using a FastDNA spin kit (MP Biochemicals, LLC) following the manufacturer’s instructions. DNA concentration was measured by Qubit 2.0 fluorometer. PCR primers 505F (5′-GTGCCAGC(A/C)GCCGCGGTAA-3′) and 909R (5′-GGACTACHVGGGTWTCTAAT-3′) were used for amplification of bacterial 16S rRNA genes (41). ITS3 (5′-GCATCGATGAAGAACGCAGC-3′) and ITS4 (5′-TCCTCCGCTTATTGATATGC-3′) were applied for amplification of the fungal ITS region (42). The amplicon libraries were generated with the TruSeq DNA PCR-free sample preparation kit. PCR conditions for the bacterial 16S rRNA gene and ITS regions were as described previously (43). The PCR products were purified with the AMPure XP system and sequenced on an Illumina MiSeq PE 150 platform (Novogene, Tianjin, China). Barcodes and primers were deleted using FLASH (version 1.2.7, http://ccb.jhu.edu/software/FLASH/). Quality control was performed by removing singletons and chimeras. Effective tags were clustered into operational taxonomic units (OTUs) with a cutoff of 97% sequence similarity in UPARSE (version 7.0.1001). Bacterial and fungal OTU annotation was performed by searching against SILVA 138 and UNITE (version 7.0) databases, respectively (44, 45). Reads were normalized to 40,000 reads in each sample for subsequent analysis. OTUs that were unidentified at the kingdom level or assigned to archaea were filtered for further analysis.

### Metagenomic sequencing.

Sample aliquots of total DNA (mentioned above) were used for preparation of metagenomics libraries. Sequencing was done on an Illumina Hiseq 2000 platform (Illumina, Inc., San Diego, CA, USA) with 2 × 150-bp paired-end reads. Data analysis was carried out using the pipeline described previously (29). Quality control was performed by removal of low-quality reads and undetermined bases relying on a minimum Q score of 30 in Trimmomatic (46). A total of 722.6 GB of clean reads were obtained. High-quality sequences in each of the 30 samples were individually *de novo* assembled into contigs by Megahit assembler (-k-min 21 -k-max 141 -k-step 12) (47). Protein-encoding genes were queried in Prokka (version 1.14.6) (48) and BLAST against the NCBI non-redundant (nr) protein database using Diamond with default settings (49). Gene profiles of the 30 samples were obtained using the KEGG database in MEGAN6 Ultimate Edition (version 6.20.5) (50, 51). Genes involved in phosphorus metabolism were queried by KEGG ortholog (KO) numbers based on published literature (13, 52). Gene abundances were rarefied to the smallest number of sequences among the metagenomic samples. The *gcd* gene sequences were extracted from both metagenomics data sets and MAGs by performing queries against the KEGG database using the KEGG ortholog no. K00117. This EC number includes both membrane-bound PQQGCD (EC 1.1.5.2) and soluble PQQGCD (EC 1.1.99.35). InterProScan was used to differentiate gene-driven amino acid sequences of membrane-bound *gcd* and soluble *gcd* (13, 53). Soluble *gcd* is not involved in inorganic phosphate solubilization and therefore was excluded from further analysis. Using this approach, a total of 455 PQQGDH-encoding *gcd* genes were identified across the 30 metagenomic samples. To achieve the taxonomic assignment of particular KEGG-annotated genes, their sequences were extracted and blasted against NCBI’s non-redundant (nr) protein database using Diamond with default settings. MEGAN6 Ultimate Edition was used for parsing and downstream analysis of the BLAST output (54).

### Taxonomic assignment and functional annotation of MAGs.

Metagenome-assembled genomes (MAGs) were obtained using metaWrap (55). The completeness and contamination of the MAGs were assessed by CheckM (version 1.1.2) (56). Only MAGs with more than 70% completeness and less than 10% contamination were selected for further analysis. Their taxonomic classification was done with GTDB-Tk (version 1.3.0) (57). Prokka (version 1.14.6) was used to predict protein-encoding genes. The putative amino acid sequences were further subjected to a BLAST search against the NCBI-nr database using Diamond with an E value threshold of 10^−5^ (49). A maximum likelihood tree of 14 MAGs was constructed based on a concatenated alignment of 120 marker genes identified in GTDB-Tk. InterProScan was applied to differentiate between *gcd* genes encoding membrane-bound PQQGDH and soluble PQQGDH in 14 MAGs (13, 53). The phylogenetic tree was subsequently visualized by the iTol (58).

### Statistical analyses.

The R environment (version 3.6.1) was used to perform all statistical analyses (http://www.r-project.org/). Significant differences between different treatments were tested by analysis of variance (ANOVA), and the *P* value was false-discovery rate (FDR) adjusted. Microbial beta diversity, which was represented by Bray-Curtis distance, was calculated using the *vegan* package. Principal-coordinate analysis (PCoA) was performed to quantify the variance in microbial community composition and function of the five treatments. Random forest analysis was further used to evaluate contributions of soil chemical properties to microbial beta diversity by the R package *randomForest.* The importance of each predictor was evaluated by the increase in mean squared error (MSE). The significance of each predictor was determined in the R package *rfPermute*.

OTU abundances that significantly varied among the five different treatments were identified using the package *edgeR* at an FDR-corrected value of *P < *0.05. We then adopted indicator species analysis to identify the OTUs that were positively associated with specific treatments using 10^4^ permutations in the R package *indicspecies* (59). Only OTUs whose abundances significantly differed between one or more treatments were further cross-selected as microbial indicators (60). To avoid single-read OTUs, only those OTUs with a total of at least 20 reads across the 30 samples were selected for analysis. Based on microbial community composition and niche differentiation, the indicators shared by both R40 and R10 treatments were considered as representative microbial indicators for reforestation soil. Indicators shared by CK, CF, and CFM treatments were defined as the representative microbial indicators for agricultural soil. A significant correlation between microbial indicators and soil chemical properties was calculated by Spearman correlation (*P < *0.05) and visualized in the Gephi platform. The co-occurrence network was constructed to assess microbial interactions using a total of more than 20 indicator OTUs. Only robust and significant Spearman correlations between OTUs were included (ρ > 0.80, *P < *0.05). The co-occurrence network was visualized by the *igraph* package using the Frucherman Reingold layout with 10^4^ permutations. The abundance of microbial genes involved in phosphorus transformation and identified in our metagenomic data sets were Z-score transformed and visualized in the heat map by the R package *pheatmap*. The meta-analysis was conducted by collecting the results of soil phosphorus measurements and microbiome composition published for 120 agricultural soils (maize) and 72 forest soils (61, 62). The sampling sites covered various soil types and climatic zones across China. The 16S rRNA gene sequence data of the 192 soil samples were processed through the same analysis pipeline as described above.

### Data availability.

Both amplicon and metagenomic raw sequence data were deposited in the NCBI Sequence Read Archive (SRA) under project no. PRJNA727951 and PRJNA700129.
