# Supplementary figures and images for: Genome-Resolved Metagenomics Reveals Distinct Phosphorus Acquisition Strategies between Soil Microbiomes
Source: mSystems. 2022 Jan 11;7(1):e01107-21. doi: 10.1128/msystems.01107-21 (PMC8751388; doi:10.1128/msystems.01107-21)

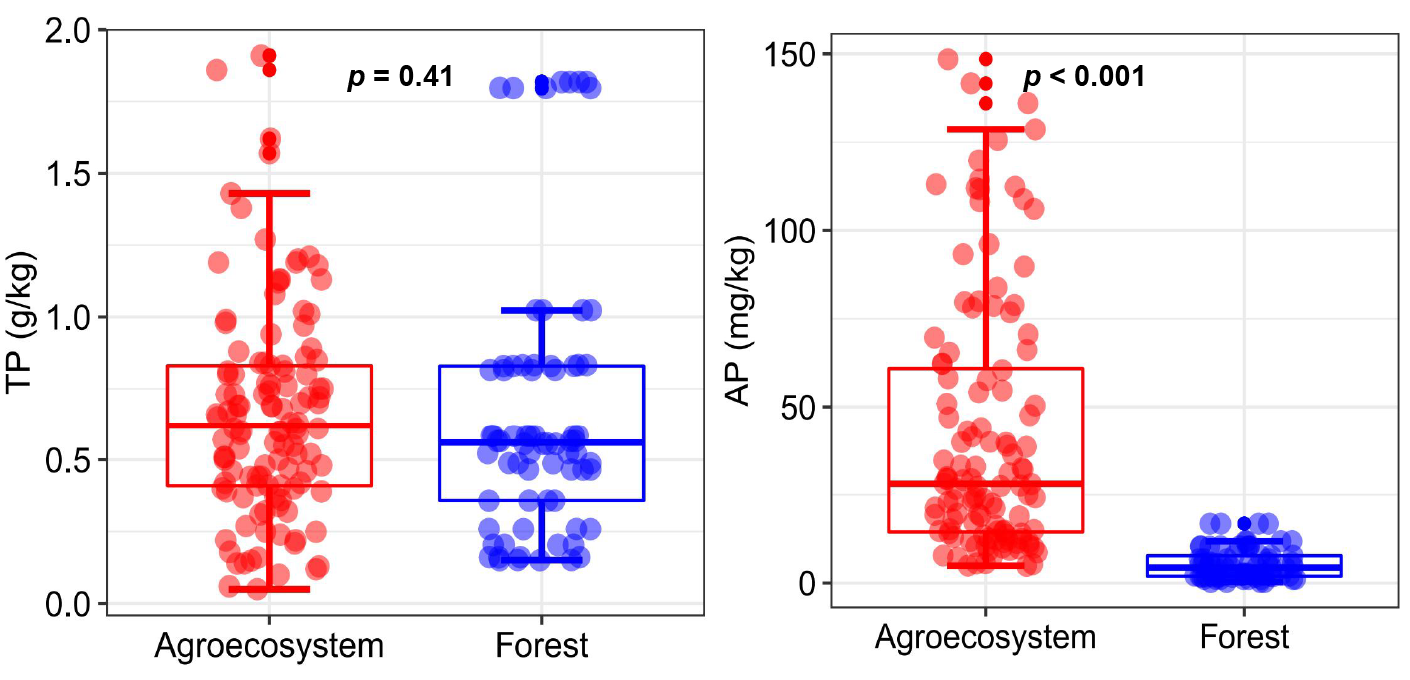

Supplement: FIG S1 [file msystems.01107-21-sf001.tif]

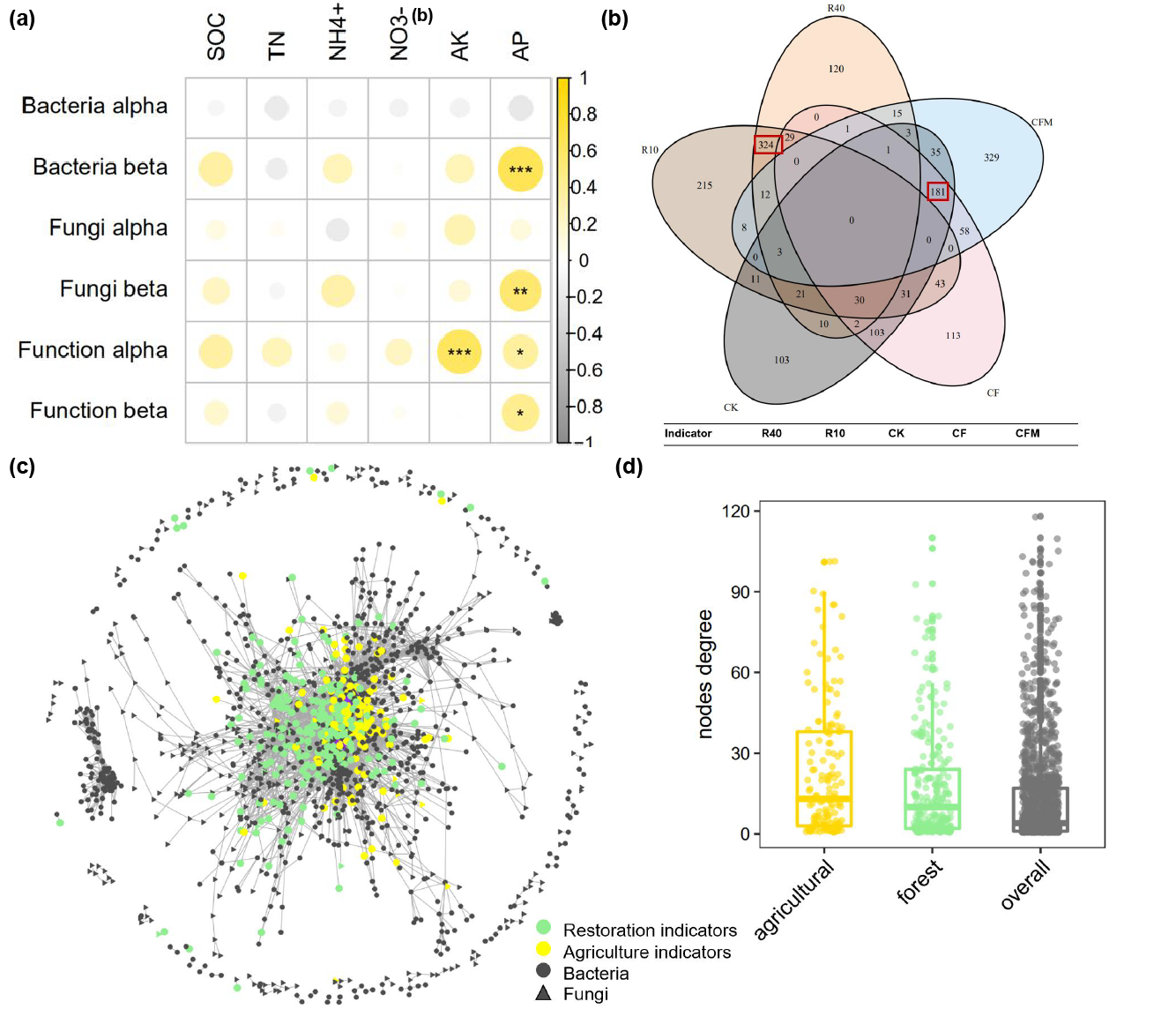

Supplement: FIG S2 [file msystems.01107-21-sf002.tif]

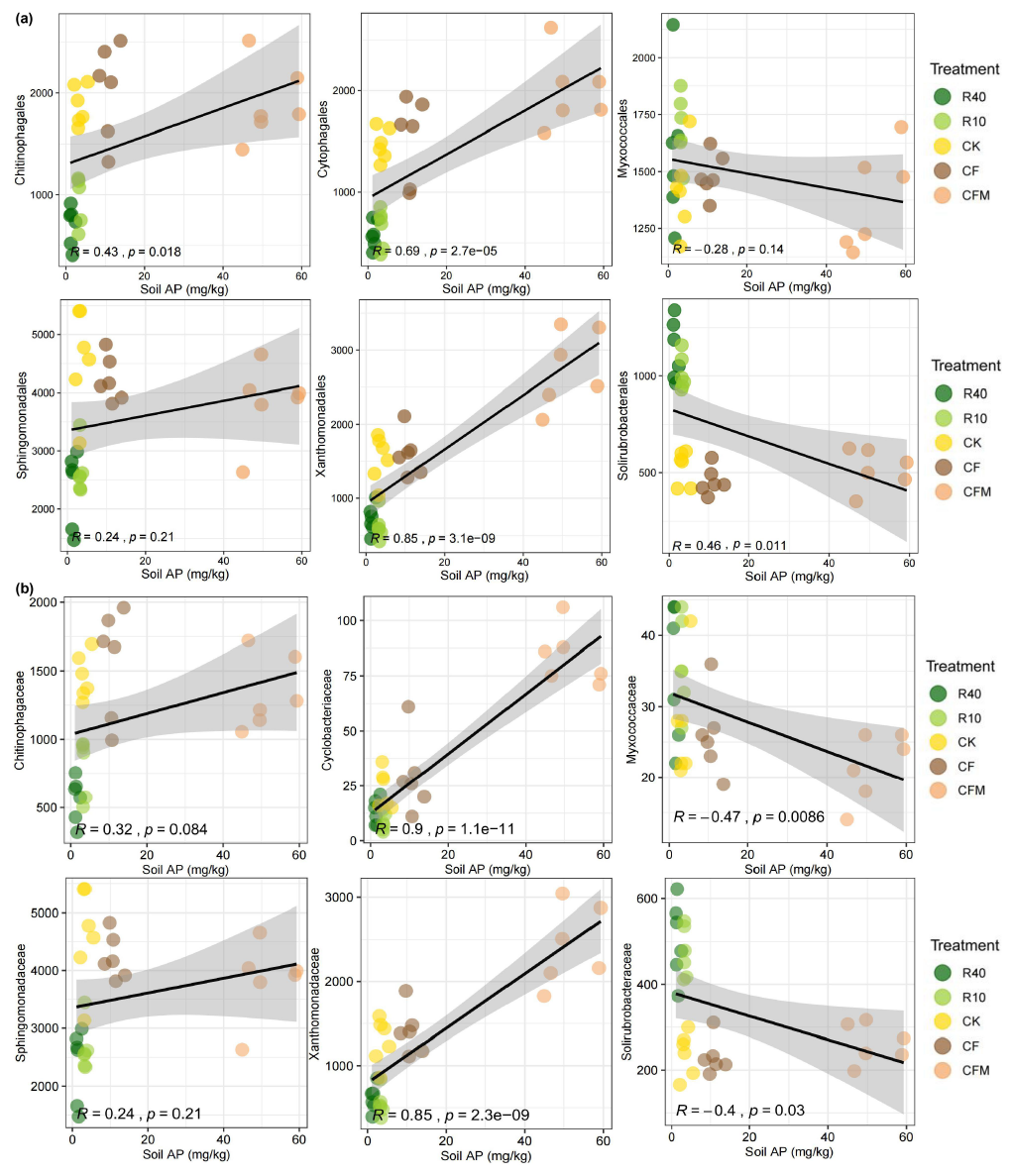

Supplement: FIG S3 [file msystems.01107-21-sf003.tif]

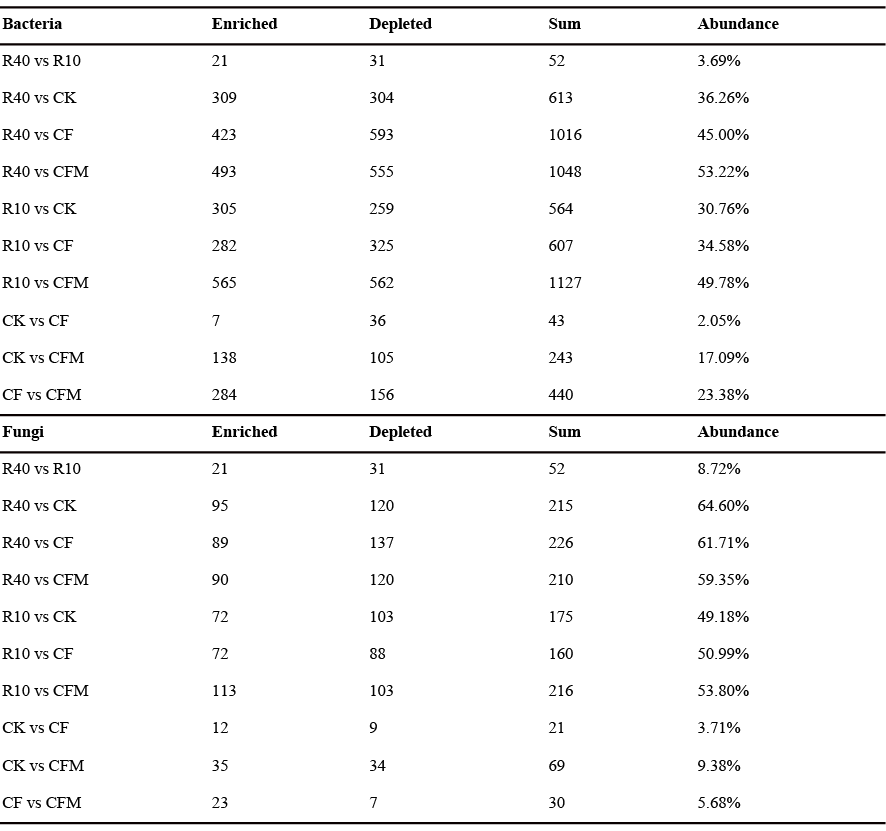

Supplement: TABLE S1 [file msystems.01107-21-st001.tif]

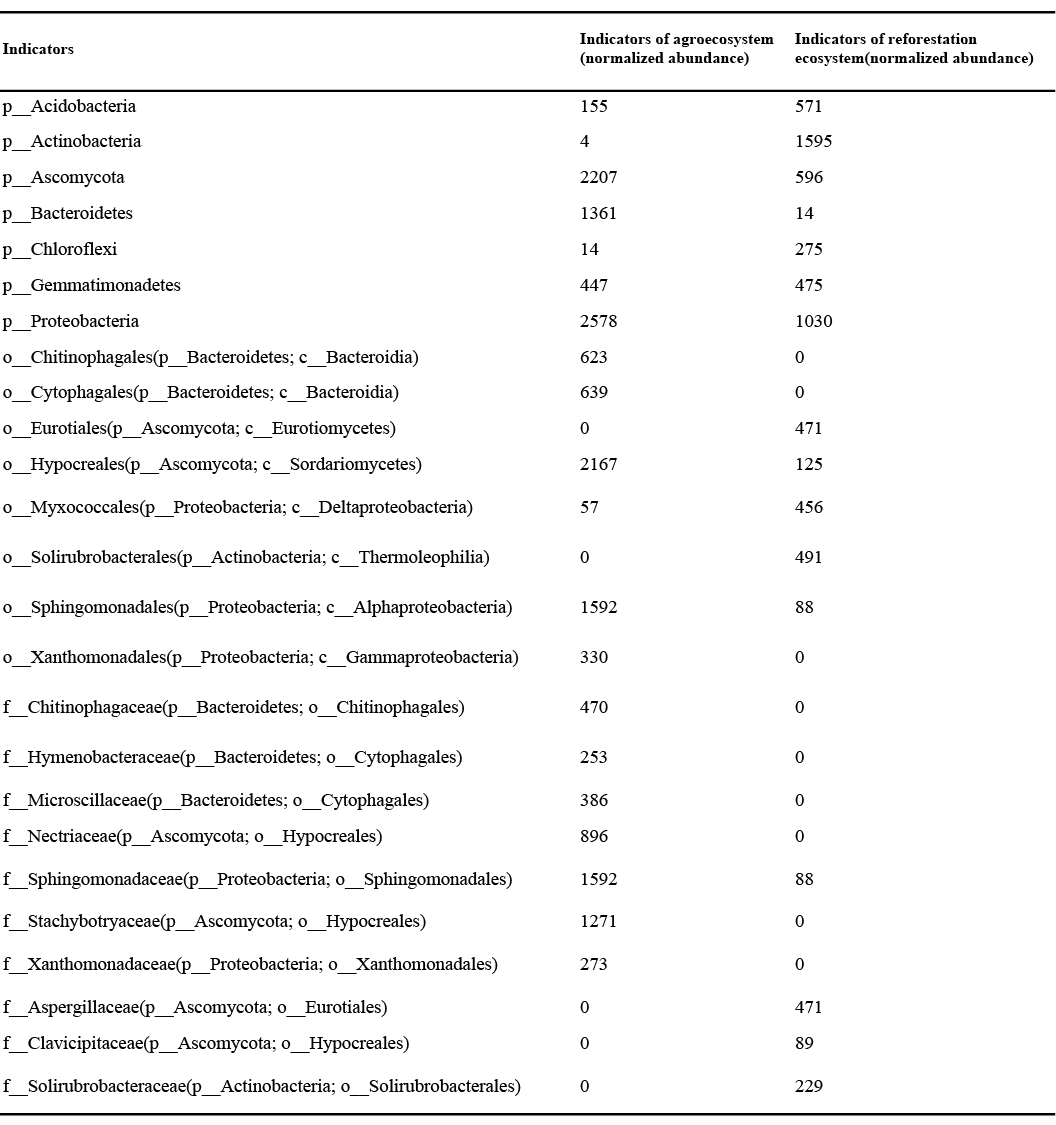

Supplement: TABLE S2 [file msystems.01107-21-st002.tif]

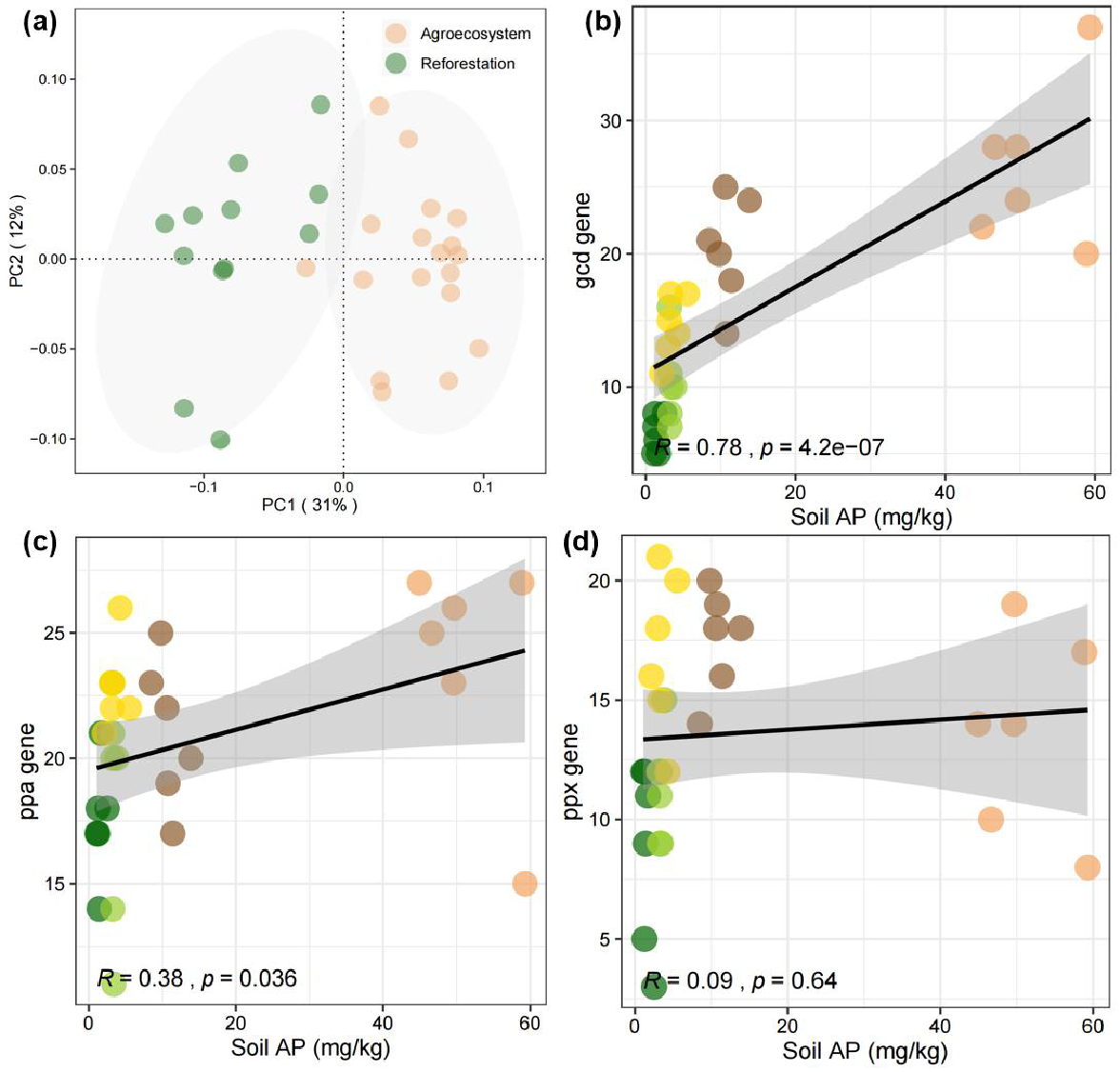

Supplement: FIG S4 [file msystems.01107-21-sf004.tif]

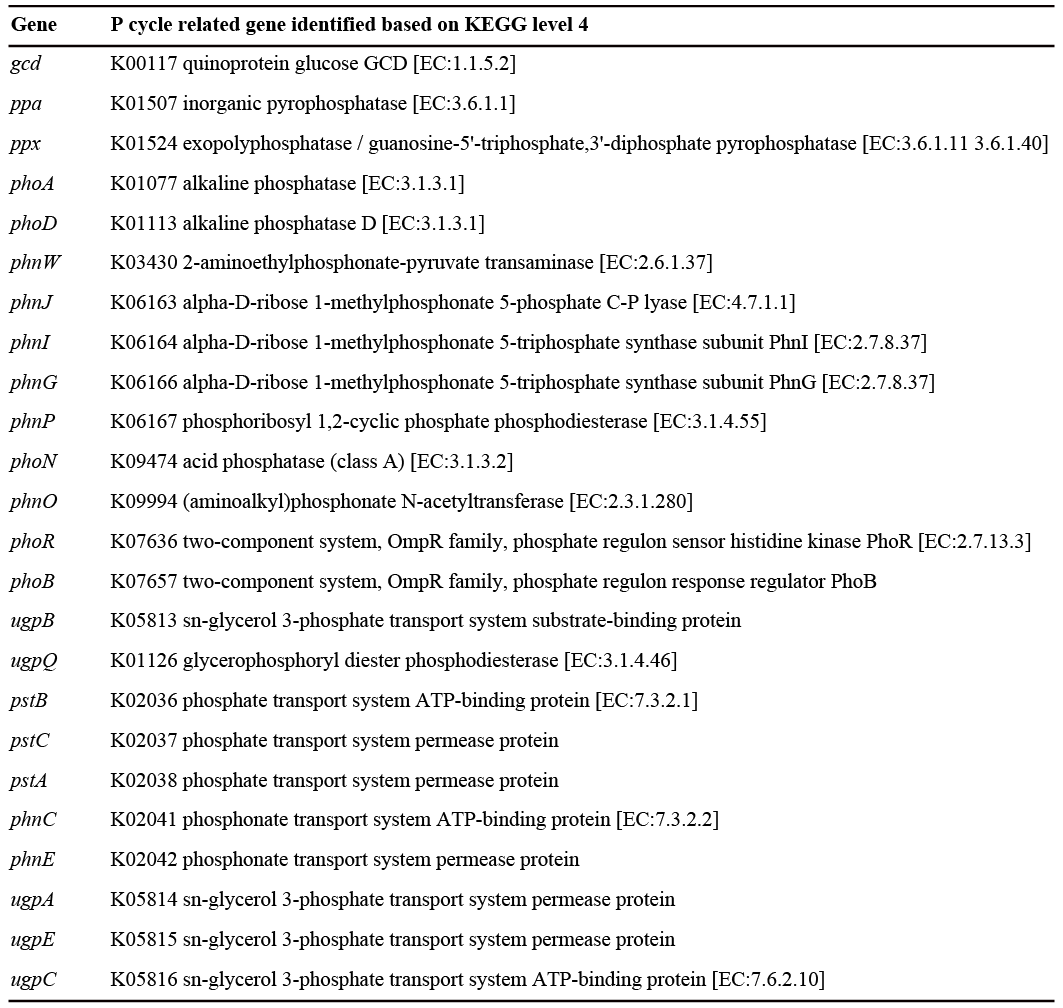

Supplement: TABLE S3 [file msystems.01107-21-st003.tif]

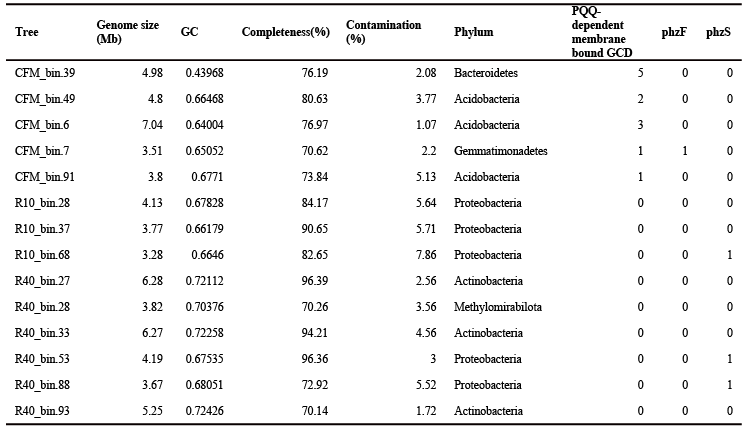

Supplement: TABLE S5 [file msystems.01107-21-st005.tif]

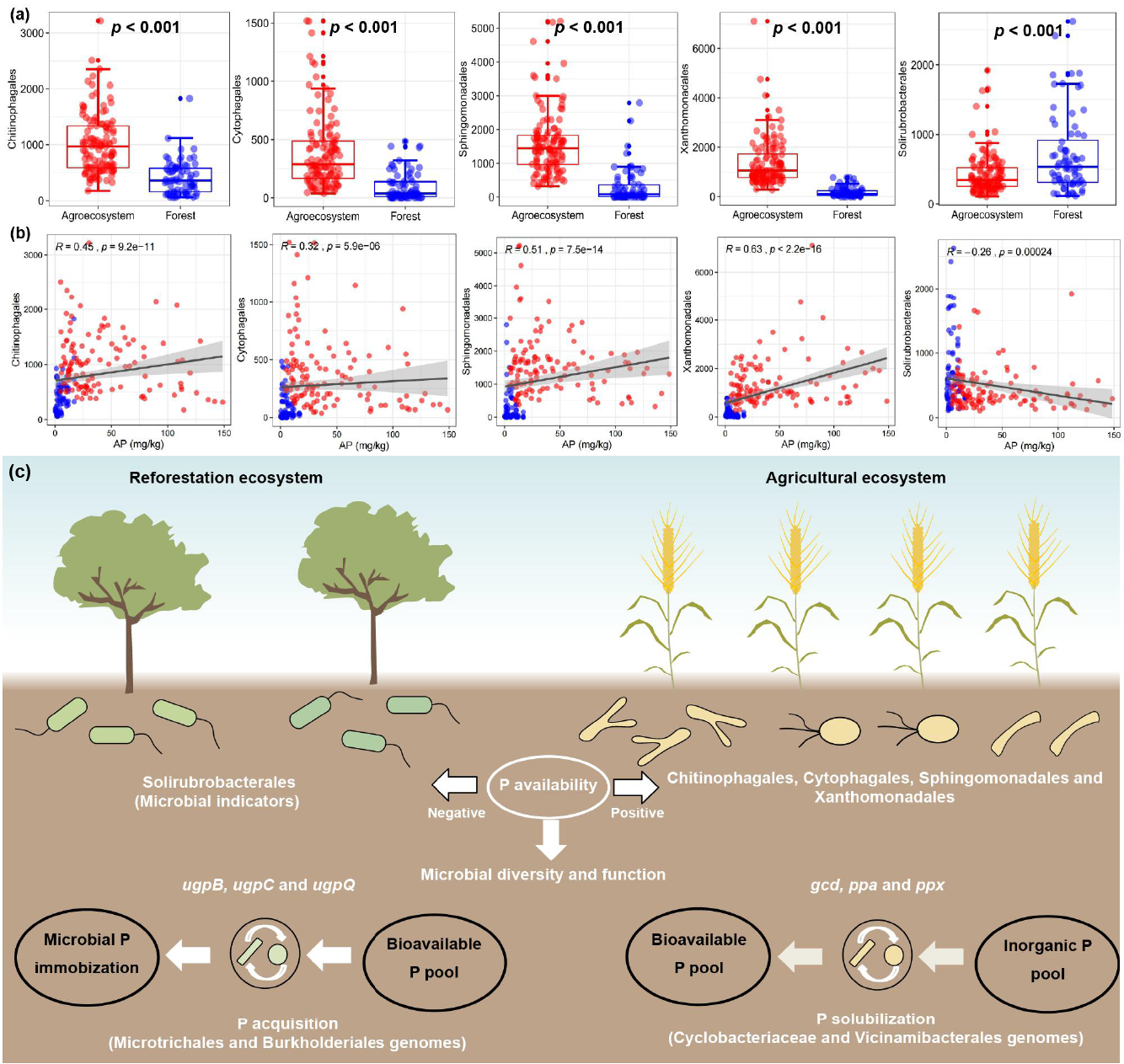

Supplement: FIG S5 [file msystems.01107-21-sf005.tif]
